# Bounded rationality, enactive problem solving, and the neuroscience of social interaction

**DOI:** 10.3389/fpsyg.2023.1152866

**Published:** 2023-05-18

**Authors:** Riccardo Viale, Shaun Gallagher, Vittorio Gallese

**Affiliations:** ^1^Department of Economics and BIB-Ciseps, University of Milano-Bicocca, Milan, Italy; ^2^Cognitive Insights Team, Herbert Simon Society, Turin, Italy; ^3^Department of Philosophy, University of Memphis, Memphis, TN, United States; ^4^SOLA, University of Wollongong, Wollongong, NSW, Australia; ^5^Department of Medicine and Surgery, Unit of Neuroscience, University of Parma, Parma, Italy; ^6^Italian Academy for Advanced Studies, Columbia University, New York, NY, United States

**Keywords:** bounded rationality, embodied cognition, problem solving, decision making, enaction

## Abstract

This article aims to show that there is an alternative way to explain human action with respect to the bottlenecks of the psychology of decision making. The empirical study of human behaviour from mid-20th century to date has mainly developed by looking at a normative model of decision making. In particular Subjective Expected Utility (SEU) decision making, which stems from the subjective expected utility theory of Savage (1954) that itself extended the analysis by Von Neumann and Morgenstern (1944). On this view, the cognitive psychology of decision making precisely reflects the conceptual structure of formal decision theory. This article shows that there is an alternative way to understand decision making by recovering Newell and Simon’s account of problem solving, developed in the framework of bounded rationality, and inserting it into the more recent research program of embodied cognition. Herbert Simon emphasized the importance of problem solving and differentiated it from decision making, which he considered a phase downstream of the former. Moreover according to Simon the centre of gravity of the rationality of the action lies in the ability to adapt. And the centre of gravity of adaptation is not so much in the internal environment of the actor as in the pragmatic external environment. The behaviour adapts to external purposes and reveals those characteristics of the system that limit its adaptation. According to Simon (1981), in fact, environmental feedback is the most effective factor in modelling human actions in solving a problem. In addition, his notion of *problem space* signifies the possible situations to be searched in order to find that situation which corresponds to the solution. Using the language of embodied cognition, the notion of problem space is about the possible solutions that are enacted in relation to environmental affordances. The correspondence between action and the solution of a problem conceptually bypasses the analytic phase of the decision and limits the role of symbolic representation. In solving any problem, the search for the solution corresponds to acting in ways that involve recursive feedback processes leading up to the final action. From this point of view, the new term *enactive problem solving* summarizes this fusion between bounded and embodied cognition. That problem solving involves bounded cognition means that it is through the problem solver’s enactive interaction with environmental affordances, and especially social affordances that it is possible to construct the processes required for arriving at a solution. Lastly the concept of *enactive problem solving* is also able to explain the mechanisms underlying the adaptive heuristics of rational ecology. Its adaptive function is effective both in practical and motor tasks as well as in abstract and symbolic ones.

## 1. Introduction

We begin with a brief background history of Subjective Expected Utility (SEU) decision making. On this view, the cognitive psychology of decision making precisely reflects the conceptual structure of formal decision theory. In relation to this structure and the normative component derived from it, empirical research in the cognitive psychology of decision making has been developing since the 1950s. This article shows that there is an alternative to this view that recovers Newell and Simon’s bounded rationality account of problem solving and integrates it into the recently developed research program of embodied cognition. The role of embodied cognition is fundamental in the pragmatic activity of problem solving. It is through the problem solver’s enactive interaction with environmental affordances, and especially social affordances that it is possible to construct the processes required for arriving at a solution. In this respect, the concept of bounded rationality is reframed in terms of embodied cognition.

## 2. Bounded rationality is bounded by the decision making programme

The empirical study of human behaviour from the mid-20th century to date has mainly developed by looking at a normative model of decision making. In particular Subjective Expected Utility (SEU) decision making, which stems from the subjective expected utility theory of Savage (1954) that itself extended the analysis of Von Neumann and Morgenstern (1944).^1^

In decision theory, the von Neumann–Morgenstern utility theorem^2^ shows that under certain axioms of rational behaviour, such as completeness and transitivity, a decision maker faced with risky (probabilistic) outcomes of different choices will behave as if he or she is maximizing the expected value of some function defined over the potential outcomes at some specified point in the future. The theory recommends which option rational individuals should choose in a complex situation, based on their risk appetite and preferences. The theory of subjective expected utility combines two concepts: first, a personal utility function, and second a personal probability distribution (usually based on Bayesian probability theory).^3^

The concepts used to define the decision are therefore information about the world; the risk related to outcomes and consequences; preferences over alternatives; the relative utilities on the consequences; and, finally, the computation to maximize the subjective expected utility. Even if in formal decision theory no explicit reference is made to the actual mental and psychological characteristics of the decision maker, in fact the concepts that define decision can be mapped onto psychological processes, such as the processing of external perceptual incoming inputs or internal mnemonic inputs, mental representations of the states of the world on the basis of information, hedonic evaluations^4^ of the states of the world, and deductive and probabilistic computation on the possible decisions to be implemented on the basis of hedonic evaluations (Viale, 2023a).

On this view, the cognitive psychology of decision making precisely reflects the conceptual structure of formal decision theory. In relation to this structure and the normative component derived from it, empirical research in the cognitive psychology of decision making has been developing since the 1950s. Weiss and Shateau (2021), highlight that in the 1950s Edwards (1992), the founder of the psychology of decision making, began to carry out laboratory experiments to unravel the way in which people actually decide. His experiments, which became the reference of subsequent generations and in particular of Daniel Kahneman and Amos Tversky’s Heuristics and Biases program, have two fundamental characteristics: firstly, the provisions of the SEU are set as a normative reference, and the experimental work has the aim of evaluating when and how the human decision maker deviates from the requirements of the SEU. Ultimately, the aim is to discover the irrational components in the decision which constitutes its bounded rationality.^5^ Secondly, the experiments are not carried out in the real decision-making contexts of everyday life, but in abstract situations of games, gamblings, bets and lotteries. In these abstract experimental situations, characterized by risk, the informative characteristics typical of the real environment - such as uncertainty, complexity, poor definition of data, instability of phenomena, dynamic and interactive change with the decision maker, and so on - are entirely absent (Viale, 2023a,b).

This situation is highlighted by Lejarraga and Hertwig (2021). Psychological experimentation on decision making,^6^ particularly within the Heuristics and Biases program, uses experiments that represent descriptions of statistical events on which a probabilistic judgment is asked. These are generally descriptions of games, bets and lotteries and other situations that do not correspond to the decision-making reality and the natural habitat of the individual and which, above all, exclude learning. The experiments in the Heuristics and Biases program do not fulfill the Brunswik (1943, 1952, 1955, 1956) requirements for psychological experiments. Since the psychological processes are adapted in a Darwinian sense to the environments in which they function, then the stimuli should be sampled from the organism’s natural ecology to be representative of the population of the stimuli to which the organism has adapted and to which the experimenter wishes to generalize. Therefore, an experiment should correspond to an experience and not to a description; it should be continuous and not discrete; and it should be ecological, normal and representative, and not abstract and unreal.

Furthermore, the highly artificial experimental protocols of the Heuristics and Biases program are frequently based on one-shot situations.^7^ They do not correspond to how people learn and decide in a step-by-step manner, thus adapting to the demands of the environment. There is no room for people to observe, correct and craft their responses as experience accumulates. There is no space for feedback, repetition or opportunities to change. Consequently, conclusions about the irrationality of the human mind have been based on artificial experimental protocols (Viale, 2023a).

In summary, the psychology of decision making reflects the conceptual *a priori* structure of SEU theory. The formal concepts used to define decision making are mapped onto psychological processes involving perception, memory, mental representations of the states of the world, hedonic evaluations, and deductive and probabilistic computation on the possible decisions to be implemented on the basis of hedonic evaluations. The limits of this research tradition are evident in relation to bounded rationality (Viale, 2023a,b):

- The provisions of the SEU are set as a normative reference, and the experimental work has the aim of evaluating when and how the human decision maker deviates from the requirements of the SEU. Ultimately, the aim is to discover the irrational performances in the decision.
- Secondly, the experiments are not carried out in the real decision-making contexts of everyday life, but in an abstract one of games, bets and lotteries. In these abstract experimental situations, characterized by risk, the informative characteristics typical of the real environment - such as uncertainty, complexity, poor definition of data, instability of phenomena, dynamic and interactive change with the decision maker, and so on - are entirely absent. Accordingly, such experiments do not fulfil the Brunswik ecological requirements.

## 3. Problem solving as an alternative programme

When Herbert Simon introduced the arguments about the limits of rationality (Simon, 1947), he did so by referring to behaviour in public administration and industrial organizations. Unlike consumer behaviour whose rationality is evaluated in relation to the SEU theory, behaviour in organizations is evaluated above all at a routine or problem-solving level. The routines of the different hierarchical levels are the main way in which problems related to the processing of information complexity and uncertainty of the external environment are solved. But it is above all in solving new problems that Simon characterizes non-routine behaviour. Depending on successful problem solving in areas such as Research & Development, marketing, distribution, human resources, finance, etc. an organization may or may not survive. The problem-solving behaviours, that can subsequently become routines, express the adaptive capacity of an organization in a more or less competitive environment. The decision-making model linked to the SEU theory does not seem relevant to the organizational context and does not seem to be at the origin of the concept of Bounded Rationality (Viale, 2023a,b).

Simon (1978) emphasizes the importance of problem solving and differentiates it from decision making, which he considers a phase downstream of the former. In fact, Simon’s research in AI, economic and organizational theory is almost entirely dedicated to problem solving that seems to absorb the evaluation and judgment phase (Viale, 2023c). In dealing with a task, humans have to frame problems, set goals and develop alternatives. Evaluations and judgments about the future effects of the choice are the optional final stages of the cognitive activity.^8^ This is particularly true when the task is an ill-structured problem. When a problem is complex, it has ambiguous goals and shifting problem formulations; here cognitive success is characterized mainly by setting goals and designing actions. Simon offers the example of design-related problems:

[T]he work of architects offers a good example of what is involved in solving ill-structured problems. An architect begins with some very general specifications of what is wanted by a client. The initial goals are modified and substantially elaborated as the architect proceeds with the task. Initial design ideas, recorded in drawings and diagrams, themselves suggest new criteria, new possibilities, and new requirements. Throughout the whole process of design, the emerging conception provides continual feedback that reminds the architect of additional considerations that need to be taken into account (Simon, 1986, p. 15).

Most of the problems in corporate strategy or governmental policy are as ill-structured as problems of architectural and engineering design or scientific activity. Reducing cognitive success to predictive ability (Schurz and Hertwig, 2019) seems to branch from the decision-making tradition and in particular from SEU theory. The latter deals solely with analytic judgements and choices, and it is not interested in how to frame problems, set goals and develop a suitable course of action (Viale, 2021, 2023a,b). In the SEU approach empirical phenomena lose their epistemic and material identity and are symbolically deconstructed and manipulated as cues with only statistical meaning (tallied, weighted, sequenced and ordered) (Felin and Koenderink, 2022).

In contrast, cognitive success in most human activities is based precisely on the successful completion of the phases of problem-solving described by Simon. Problem-solving is not the computation of a decision based on an analytical prediction activity performed on data coming from deconstructed empirical phenomena, but rather a pragmatic recursive process made up of many attempts and related positive or negative feedback from the environment.

Simon’s approach to problem solving highlights the influence of American pragmatism, and in particular of Dewey (1910), Peirce (1931), and James (1890), on his work. For the pragmatists, the centre of gravity of the rationality of action lies in the ability to adapt. And the centre of gravity of adaptation is not so much in the internal environment of the actor, that is, in his or her cognitive characteristics, as in the pragmatic external environment. Simon and Newell write: “For a system to be adaptive means that it is capable of grappling with whatever task environment confronts it. Hence, to the extent that a system is adaptive, its behaviour is determined by the demands of the task environment rather than by its own internal characteristics. Only when the environment stresses [the system’s] capacities along some dimension - presses its performance to the limit - do we discover what those capabilities and limits are, and are we able to measure some of their parameters” (Newell and Simon, 1971, p. 149).

## 4. Enactive problem solving and 4E cognition

In this section we argue that the role of embodied cognition is fundamental in this pragmatic activity. We take embodied cognition in a broad sense to include what has been termed 4E (embodied, embedded, extended and enactive) cognition (Newen et al., 2018). On this view, the body’s neural and extra-neural processes, as well its mode of coupling with the environment, and the environmental feedback that results, play important roles in cognition. Similar to Simon’s approach, 4E cognition has philosophical roots in pragmatism (see especially Gallagher, 2017; Crippen and Schulkin, 2020), but also incorporates insights from phenomenology, analytic philosophy of mind, developmental and experimental psychology and the neurosciences.

Wilson (2002) outlined a set of principles embraced by most proponents of embodied or 4E cognition.

1. cognition is situated
2. cognition is time-pressured
3. we off-load cognitive work onto the environment
4. the environment is part of the cognitive system
5. cognition is for action
6. cognition (in both basic and higher-order forms) is based on embodied processes

Proponents of 4E approaches, however, vary in what they emphasize as explanatory for cognition. The body can play different roles in shaping cognition. Non-neural bodily processes are sometimes thought to shape sensory input prior to, and motor output subsequent to central or neural manipulations (e.g., Chiel and Beer, 1997). According to proponents of extended cognition minimal, action-oriented representations add further complexity (Clark, 1997a; Wheeler, 2005). Enactive approaches emphasize the idea that the body is dynamically coupled to the environment is important ways (Di Paolo, 2005; Thompson, 2007); they point not only to sensorimotor contingencies (where specific kinds of movement change perceptual input) (O’Regan and Noë, 2001), but also to bodily affectivity and emotion (Gallese, 2003; Stapleton, 2013; Colombetti, 2014) as playing a nonrepresentational role in cognition. Embedded and enactive approaches emphasize action affordances that are body- and skill-relative (Chemero, 2009). More generally, most theorists of embodied cognition hold that these ideas help to shift the ground away from orthodox, purely computational cognitive science, which clearly informs the cognitive psychology of decision making. In this respect, it’s not just the internal processes of the mind or brain, but the brain–body-environment system that is the unit of explanation.

Relevant to the idea of problem solving, there is general agreement that the environment scaffolds our cognitive processes, and that our engagement with the environmental structure, and environmental features, including external props and devices, can shift cognitive load. Already, within the scope of Simon’s own work it’s clear that only through the enactive interaction between problem solver and environmental affordances is it possible to construct a solution. The metaphor of the ant on the beach (Simon, 1981) is illuminating: imagine an ant walking on a beach. Now let us say you wanted to understand why the ant is walking in the particular path that it is. In Simon’s parable, you cannot understand the ant’s behaviour just by looking at the ant: “Viewed as a geometric figure, the ant’s path is irregular, complex, hard to describe. But its complexity is really a complexity in the surface of the beach, not a complexity in the ant” (Simon, 1981, p. 80). In other words, to predict the path of the ant, we have to consider the effects of the beach – the context that the ant is operating in. The message is clear: we cannot study what individuals want, need or value detached from the context of the environment that they are in. That environment shapes and influences their behaviour. In this example, the *procedural rationality* of the ant (finding a suitable behaviour on the beach) requires its *substantial rationality* (the adaptivity to the irregularity of the beach).

From this metaphor Simon derives a philosophical principle very much in tune with the broad sense of 4E cognition^9^: “A man considered as a system capable of having a behaviour is very simple. The apparent complexity of his behaviour over time is largely a reflection of the complexity of the environment in which he finds himself” (Simon, 1981, p. 81). The behaviour adapts to external purposes and reveals those characteristics of the system that limit its adaptation.

When agents coordinate their activity with environmental resources such as external artifacts, cognitive processes may be productively constrained or enabled by objective features, or enhanced by the affordances on offer. Examples include using written notes to reduce demands on working memory, setting a timer as a reminder to do something, using a map, or the surrounding landscape to assist in navigation, or, since the environment is not just physical, but also social, asking another person for directions (Gallagher, in press).

For the idea of enactive problem solving, however, it is important to emphasize two things. First, the relational nature of affordances. It is not just the environment that constrains behaviour; it is also the body’s morphology and motor possibilities, and the agent’s past experience and skill level that will define what counts as an affordance. The way in which the body couples (or can couple) to the environment, will delineate the set of possibilities or solutions available to the agent. Likewise, affordances can also be limited by an agent’s affective processes, emotional states, and moods. It is sometimes not just what “I can” do (given my skill level and what the environment affords), but what “I feel like (or do not feel like)” doing (given my emotional state).

Second, as the pragmatists pointed out, the environment is not just the physical surroundings; it’s also social and cultural and characterized by normative structures. As Gibson (1979) indicated, affordances can be social. Enactive problem solving also highlights the important role of social and intersubjective interactions (De Jaegher, 2018). Again, it’s not only what “I can” do, but also what “I cannot” (or “I ought not”) do given normative or institutional constraints, as well as cultural factors that have to do with, for example, gender and race. These are larger issues that range from understanding how dyadic interactions shape our developing skills, to how institutional factors can either enable or constrain our social interactions.

It is also the case that cultural practices can determine the way in which the environment is represented, thereby changing our ability to interact with it. Think of how much arithmetic was simplified by transitioning from Roman to Arabic numerals and to positional notation. The success of the Arabic number system was dictated by the positive pragmatic aspects it delivered in our ability to efficiently represent the world in quantitative terms.^10^ In other words, it was the retroactive adaptation that allowed the Arabic number system to prevail. Embodied processes are primitive and original in the cultural development of mathematical calculus and geometry. In a set of well-known experiments, Goldin-Meadow et al. (2001) showed that hand gesture may add to or supplement mathematical thinking. Specifically, children perform better on math problems when they are allowed to use gestures. In addition, Lakoff and Nunez (2000, p. 28) argue that mathematical reasoning builds on innate abilities for “subitizing,” i.e., discriminating, at a glance, between there being one, or two, or three objects in one’s visual field, and on basic embodied processes involving “spatial relations, groupings, small quantities, motions, distribution of things in space, changes, bodily orientations, basic manipulations of objects (e.g., rotating and stretching), iterated actions, and so on.” Thus, the concept of a set is derived from perception of a collection of objects in a spatial area; recursion builds upon repeated action; derivatives (in calculus) make use of concepts of motion, boundary, etc. (Lakoff and Nunez, 2000, pp. 28–29).^11^ Likewise, Saunders Mac Lane (1981) provides “examples of advances in mathematics inspired by bodily and socially embedded practices: counting leading to arithmetic and number theory; measuring to calculus; shaping to geometry; architectural formation to symmetry; estimating to probability; moving to mechanics and dynamics; grouping to set theory and combinatorics” (Gallagher, 2017, p. 209). All such practices involve environmental feedback as an essential part of the process.

According to Simon (1981), in fact, environmental feedback is the most effective resource for modelling human actions in solving a problem. Design activity is shaped by the logic of complex feedback. A purpose is followed in the design, which is to solve a given problem (e.g., design a smooth urban plan for the regulation of road traffic), and when you think you have reached it, feedback is generated (e.g., from the political, social and geographical environment) that introduces a new, unforeseen purpose (e.g., energy saving constraints). This leads to reworking the design and generating new retroactive effects. The same selectivity in the solution of a problem is based on feedback from the environment (Simon, 1981, p. 218).

Newell and Simon (1971) propose the notion of the problem space. They write (p.150): a “problem space is about the possible situations to be searched in order to find that situation which corresponds to the solution.” The concept of problem space can easily be characterized in terms of enactive interaction and coupling with environmental affordances. A problem space is equivalent to the possible solutions that can be enacted given the landscape of affordances (Rietveld and Kiverstein, 2014). Some of the resources that define a solution will come from past experience and one’s skill set; some others from the consequences of the actions that have been attempted in pursuit of the solution. The actions leading to the solution manipulate the world in a recursive feedback process, whereas processes of forecasting, which often lead the problem solver into a dead end, have limited importance. In fact, for Simon (1981, p. 231) the distinction between “state description” that describes the world as it is and “process description” that characterizes the steps in manipulating the world to achieve the desired end is important. To use another Simonian figure: given a certain dish, the aim is to find the corresponding recipe (Simon, 1981, p. 232). This research takes place through successive actions with phenomenological/sensory-motor feedback(taste, smell, texture) selectively directing us towards the final result. And, we may add, this happens not only when the problem is not well structured, as in the case in which we do not have the recipe data, but also when we know the necessary ingredients.

The correspondence between action and the solution of a problem conceptually bypasses the analytic phase of the decision and limits the role of symbolic representation. The decision-making model based on SEU theory does not correspond to the empirical reality of individual action. In solving any problem, whether opening a door, running to catch a falling ball, replacing a car tyre, calculating for a financial investment, solving tests and puzzles or negotiating with a competitor, the search for the solution corresponds to acting in the sense of *wide* and *strong* embodied cognition, including the idea of a recursive feedback process leading up to the final action. From this point of view, the concept of ‘enactive problem solving’ summarizes the integration of multiple factors and could well represent the complexity of the phenomenon (Viale, 2023a).

The importance of the embodied aspects of human cognition that emerge from the concept of enactive problem solving can also be demonstrated in the actions generated by the simple heuristics studied within the ecological rationality program (Gigerenzer, Todd, and ABC Group, 1999; Gallese et al., 2021). Ecological rationality represents the direct development of bounded rationality. Most ecological rationality heuristics have to do nominally with decision making, but in actuality are often enactive problem solving mechanisms, and they can be analysed in terms of embodied cognition. In support of this thesis, consider the main mental abilities that heuristics use in their activation. The core mental capacities exploited by the building blocks of simple heuristics include *recognition memory*, *frequency monitoring* and additionally, three typical embodied cognition capacities: visual *object tracking*, *emotion* and *imitation* (Hertwig and Herzog, 2009; Gigerenzer and Gassamaier, 2011; Hertwig and Hoffrage, 2011).

Gigerenzer (2022) writes that he “reserves the term ‘embodied heuristics’ for rules that require specific sensory and/or motor abilities to be executed, not for rules that merely simplify calculations” (Gigerenzer, 2022). In reality, the very capacity of frequency monitoring seems to reflect a dimension of embodiment. A confirmation of this comes from the considerations of Lejarraga and Hertwig (2021) on the importance of experimental protocols that include learning and experience. Why are the heuristics and biases experimental protocols in behavioural decision research that rely on described scenarios rather than learning and experience able to cause so many biases? Which qualities of experience make it different from description and thus potentially foster statistical intuitions? Lejarraga and Hertwig write: “A learner experiencing a sequence of events may, for instance, simultaneously receive sensory and motor feedback (potentially triggering affective or motivational processes); obtain temporal, structural, and sample size information” (Lejarraga and Hertwig, 2021, p. 557). In other words, the ability to respond correctly in repeated and experience-based statistical tests is derived from the adaptive role of the sensorimotor and affective feedback-loop associated with the task. Thus, enactive problem solving is also able to explain the mechanisms underlying the adaptive heuristics of rational ecology. Its adaptive function seems effective both in practical and motor tasks as well as in abstract and symbolic ones.

## 5. The inside story

In 4E approaches much of the emphasis falls on embodied and environmental processes. Perhaps this is a reaction to the overemphasis in classic computational cognitive science that emphasizes processes internal to the individual agent. 4E cognition, however, does not deny the important role of brain processes. Neural processes are dynamically coupled to non-neural bodily processes. Indeed, the explanatory model is brain–body-environment. So how should we characterize what is happening in the brain in this model, especially as it relates to affordance-related processes and social cognition and interaction?

In regard to the latter, we note that primates learn from others’ behaviour and base their decisions also on the prediction of others’ choices. The discovery of ‘mirror neurons’ in macaque monkeys (Gallese et al., 1996; Rizzolatti et al., 1996), and then of similar mechanisms in humans (see Gallese et al., 2004), revealed the cognitive role of the motor system in social cognition, enabling the start of social neuroscience. The solipsistic stance of classic cognitivism, addressing the ‘problem of other minds’ by means of a disembodied computational architecture applied to a social arena populated by other cognitive monads was finally challenged, giving way to an embodied account of intersubjectivity, grounded on what the phenomenologist, Merleau-Ponty (2012), called intercorporeity. Indeed, mirror neurons reveal a new empirically founded notion of intersubjectivity connoted first and foremost as the mutual resonance of intentionally meaningful sensorimotor behaviours. We believe that these empirical findings have important bearings on decision making and problem solving by revealing their intrinsic social and embodied quality.

Thirty years of empirical research on mirror neurons have shown that the perceptual functions of the human motor system may be linked with its evolutionarily retained relevance in planning and coordinating behavioural responses coherent with the observed action of others (for a recent review, see Bonini et al., 2022; see also Bonini et al., 2023). The picture, however, is more complex than originally thought. Recent studies employing chronically implanted multiple recording devices revealed that in macaques’ lateral and mesial premotor areas, besides ‘classic’ mirror neurons there are neurons exclusively mapping the actions of others while lacking motor responses during action execution. Two recent studies are particularly relevant for issues pertaining to decision making and problem solving. Haroush and Williams (2015) used a joint-decision paradigm to study mutual decisions in macaques. The study revealed in the premotor dorsal region of the anterior cingulate cortex (dACC) neurons encoding the monkey’s own decision to cooperate intermingled with neurons encoding the opponent monkey’s decisions when they were yet unknown. The problem space, we might say, includes a reserved slot for the anticipated decisions and actions of the other agent. Another recent study by Grabenhorst et al. (2019) showed that macaques’ amygdala neurons derive object values from conspecifics’ behaviour observation (that is, from the other agents’ observed actions towards a particular object) which the system then uses to anticipate a partner monkey’s decision process. The present evidence suggests that other-related neuronal processing is co-activated with neurons encoding self-related processes in an extended network of brain areas encompassing multiple domains, from motor actions, sensations, and emotions to decisions and spatial representations, in multiple animal species. As recently proposed by Bonini et al. (2022), when individuals witness the action of others, they face different options that are known to recruit the main nodes of the human mirror neurons network: 1) faithfully imitating or emulating the observed action, 2) avoiding doing so, or 3) executing a complementary or alternative action. Both the environmental context and the contemporary state of the observer (i.e., knowledge, motivation, emotion, skill-level etc.) profoundly shape the way in which an observed action affects his/her own motor system.

As Bonini et al. (2023) recently argued, “Although the concept of shared coding grounds the history of mirror neuron literature, our recent perspective emphasizes the role of agent-based coding as a means of linking sensory information about others (i.e., *via* other-type neurons) to one’s own motor plans (i.e., self-type neurons). The inherently predictive nature of the motor and visceromotor systems, which hosts this neural machinery, enables the flexible preparation of responses to others depending on social and nonsocial contexts.” Furthermore, pioneering studies capitalizing on hyperscanning techniques that go beyond the traditional “one-brain” approach, suggest that interbrain synchronies could guide social interaction by having self-related neurons in Subject 1 controlling behaviour and, in turn, causing the activity of other-selective neurons in the brain of Subject 2, processes which finally lead to an adaptive behavioural response by activating self-related neurons (Bonini et al., 2022).

Social neuroscience, therefore, shows us that the ability to understand others as intentional agents does not exclusively depend on propositional competence, but it is in the first place dependent on the relational nature of embodied behaviour. According to this hypothesis, it is possible to directly understand others’ behaviour by means of the sensorimotor and visceromotor equivalence between what others do and what the observer can do. Thus, intercorporeity becomes the primordial source of knowledge that we have of others, informing interaction and providing an important source for evaluating problem spaces.

Empirical research has also demonstrated that the human brain is endowed with mirror mechanisms in the domain of emotions and sensations: the very same neural structures involved in the subjective experience of emotions and sensations are also active when such emotions and sensations are recognized in others. For example, witnessing someone expressing a given emotion (e.g., disgust, pain, etc.) or undergoing a given sensation (e.g., touch) recruits some of the viscero-motor (e.g., anterior insula) and sensorimotor (e.g., SII, ventral premotor cortex) brain areas activated when one experiences the same emotion or sensation, respectively. Other cortical regions, though, are exclusively recruited for one’s own and not for others’ emotions, or are activated for one’s own tactile sensation, but are actually deactivated when observing someone else’s being touched (for review, see Gallese, 2014; Gallese and Cuccio, 2015).

The recent research that we have cited thus suggests that our ability to interact with others in decision-making and problem-solving contexts is not exclusively or primarily the result of individual neurons that simply mirror others’ behaviour, but is rather based on more complex neural networks that are constituted by a variety of cell types, distributed across multiple brain areas, coupled to the body, and attuned to selective aspects of the physical and social environment. Our own planning and problem solving involve behavioural responses that depend on the behaviours of others. To put it simply, it is not the brain *per se*, but the brain–body, by means of its interactions with the world of which it is part, that enacts our cognitive capacities. The proper development of this functional architecture of brain–body-environment scaffolds the more cognitively sophisticated social cognitive (including linguistic and conceptual) abilities that constitutes our rationality (Cuccio and Gallese, 2018; Gallese and Cuccio, 2018).

## 6. Conclusion

Our brief review of Subjective Expected Utility (SEU) decision making showed some of its limitations. Newell and Simon’s approach to problem solving offers an alternative that reflects the concept of bounded cognition. We argued that this alternative fits well with some of the more recent research in embodied cognition. The role of embodied cognition and environmental feedback is fundamental in the pragmatic activity which we called enactive problem solving. This approach emphasizes bodily interaction with environmental affordances that form the problem space where solutions can be found. Explanations of such processes require an approach that emphasizes the enactive system of brain–body-environment. We highlighted the importance of specific brain processes (the mirror mechanisms) which contribute to this system in ways that facilitate complex social interactions. Only through the enactive interaction of the problem solver with environmental (including social and cultural) affordances is it possible to construct the complex solutions that characterize human design efforts.

A more detailed theory of enactive problem solving will depend to some extent on resolving some problems in the philosophy of mind and embodied cognition – basic issues that have to do with notions of information processing, computation, body-environment couplings, affordances, and how these may or may not involve representational processes of different kinds. In the meantime, linking the concepts of bounded rationality with embodied-enactive cognition should be taken as a pragmatic proposal (which itself would be an enactive problem solving approach) that could inform future experimental designs that may ultimately contribute to resolving the more theoretical problems.

## Author contributions

RV: contribution about the critique to decision making and the proposal of enactive problem solving. SG: contribution about embodied cognition and enactivism. VG: contribution about embodied simulation and mirror neurons. All authors contributed to the article and approved the submitted version.

## Conflict of interest

The authors declare that the research was conducted in the absence of any commercial or financial relationships that could be construed as a potential conflict of interest.

## Publisher’s note

All claims expressed in this article are solely those of the authors and do not necessarily represent those of their affiliated organizations, or those of the publisher, the editors and the reviewers. Any product that may be evaluated in this article, or claim that may be made by its manufacturer, is not guaranteed or endorsed by the publisher.

## Footnotes

^1^The way in which this escalation developed is discussed in detail in Mousavi and Tideman (2021).

^2^Von Neumann and Morgenstern never intended axiomatic rationality to describe what humans and other animals do or what they should do. Rather, their intention was to prove that if an individual satisfies the set of axioms, then their choice can be represented by a utility function.

^3^This theoretical model has been known for its clear and elegant structure and it is considered by some researchers to be one of “the most brilliant axiomatic theory of utility ever developed.” In contrast, assuming the probability of an event, Savage defines it in terms of preferences over acts. Savage used the states (something that is not in your control) to calculate the probability of an event. On the other hand, he used utility and intrinsic preferences to predict the outcome of the event. Savage assumed that each act and state are enough to uniquely determine an outcome. However, this assumption breaks down in the cases where the individual does not have enough information about the event. In reality Savage explicitly limited the theory to small worlds, that is, situations in which the exhaustive and mutually exclusive set of future states S and their consequences C are known.

^4^The hedonic approach to economic assessment can be used for evaluating the economic value of goods. The hedonic approach is based on the assumption that goods can be considered aggregates of different attributes, some of which, as they cannot be sold separately, do not have an individual price.

^5^Bounded Rationality was introduced by Herbert Simon (1982) to characterize the constraints of human action. As it is represented in the scissor’s metaphor there are two set of constraints: one is about the computational limitations of the mind and the other is about the complexity and uncertainty of the environment (task). The psychology of decision making and behavioural economics focussed mainly on the first cognitive set of constraints forgetting the second set.

^6^The lack of ecological soundeness applies to many areas of cognitive psychology.

^7^This is not a characteristic merely of Heuristic & Biases experiments, but of the majority of lab experiments in psychology and economics with some exceptions in repeated games experiments as in ultimatum games with multiple players. Nevertheless the perseverance to use artificial experiments protocol relies on some methodological advantages as easy control of the crucial variables, random sampling and clear task conditions.

^8^On the traditional models, problem solving includes the steps of judgement and evaluation, but does not include the stage of action. Problem solving and action, however, are both part of the phenomenon that we dub “enactive problem solving.” It is a dynamic process based on pragmatic recursive attempts and related positive or negative feedback from the environment. Constructing the meaning of one’s attempts at a solution and ultimately selecting the final solution are only possible through the problem solver’s enacting interaction with environmental affordances (Viale, 2023a).

^9^We note that although the concept of bounded rationality acknowledges the role of the environment in problem solving, it does this from an information processing perspective. In this respect bounded rationality is historically tied to a computational/cognitivist approach, rather than an embodied approach that emphasizes action-perception loops, affordances, and dynamic brain–body-environment assemblies. Some embedded and extended versions of embodied cognition can be viewed as consistent with the information processing/computational framework (e.g., Clark, 2008). Others, like the radical enactive approaches tend to reject this framework (e.g., Hutto and Myin, 2017). Our aim in this paper is not to resolve such debates in the embodied cognition literature. On our view, it remains an open question whether one can reframe bounded rationality in strict non-computational enactivist terms. In any case, Simon’s pragmatist epistemology and his account of the importance of environmental feedback in solving problems draws him closer to the enactive aspects of embodied cognition. For a contrast between extended and enactive approaches in the context of institutional economics, see Clark (1997b) and Gallagher et al. (2019).

^10^See, e.g., Overmann (2016, 2018). It is important to consider the role of materiality in defining physical affordances (found in paper and pencil, and the formation of doodles, images, and script), as well as physical practices with our hands that can lead to abstract modes of thought (Gallagher, 2017, p. 196n3; Overmann, 2017). Malafouris (2013, 2021) highlights how the fact that making straight lines was easier than making curved ones led to the development of more and more abstract forms in pictographs/ideographs. This promoted greater simplicity and speed of language production.

^11^Lakoff and Nuñez frame their analysis in terms of metaphor. For views closer to enactive approaches, see Abrahamson (2021) and Gallagher and Lindgren (2015).
